# NMR-Based Metabolomic and QMB-Based E-Nose Approaches to Evaluate the Quality and Sensory Features of Pasta Fortified with Alternative Protein Sources

**DOI:** 10.3390/molecules30163438

**Published:** 2025-08-20

**Authors:** Marika Chiossi, Diana De Santis, Margherita Modesti, Serena Ferri, Marcello Fidaleo, Francesco Buonocore, Fernando Porcelli, Esther Imperlini

**Affiliations:** Department for Innovation in Biological, Agro-Food and Forest Systems, University of Tuscia, Largo dell’Università snc, 01100 Viterbo, Italy; marikachiossi@gmail.com (M.C.); desdiana@unitus.it (D.D.S.); margherita.modesti@unitus.it (M.M.); serenaferri@unitus.it (S.F.); fidaleom@unitus.it (M.F.); fbuono@unitus.it (F.B.)

**Keywords:** ^1^H NMR spectroscopy, QMB E-nose, alternative protein sources, technological quality, metabolites, sensory profile

## Abstract

The consumption of animal- and plant-based protein food is increasing as the world population grows. Alternative protein sources that are nutritious, safe and sustainable are needed. There is a growing research interest in integrating wheat-based staple foods, such as pasta, with new ingredients that could also provide nutritional and health benefits. Despite their unquestionable nutritional value, new pasta formulations need to be evaluated in terms of technological/sensory quality. In this study, we assessed the quality and flavour of traditional egg pasta fortified with two alternative protein sources: hazelnut flour and cricket powder. It is known that a quality pasta tends to lose fewer solids during cooking. In parallel with classical evaluation of cooking and sensory characteristics, proton nuclear magnetic resonance (^1^H NMR) spectroscopy of the metabolites released during the cooking process and volatile fingerprint analysis with quartz microbalance (QMB) electronic nose (E-nose) were performed. These approaches showed results complementary to those obtained from classical quality and sensory analyses, thus demonstrating the potential of ^1^H NMR and E-nose in pasta quality assessment. Overall, the pasta fortification with cricket powder and hazelnut flour affected the matrix mobility by modulating the release of chemical components into the water during cooking and overcooking processes; moreover, it significantly altered the pasta sensory profile in terms of aroma and texture. This finding highlights the complexity of balancing technological improvement with sensory appeal in food product development.

## 1. Introduction

The human population is projected to reach 9.8 billion by 2050, leading to an increasing global food demand and a rising need for alternative protein sources [1,2,3]. To date, the protein market mainly consists of animal products derived from meat consumption, particularly in Western countries, with a growth rate of 9.1% from 2020 to 2027 [4]. On the other hand, in many undeveloped and developing countries, plant-based proteins serve as the primary protein source due to the high costs related to the production of animal proteins [5]. However, traditional animal protein production is not a sustainable process, as livestock farming utilises 67% of agricultural land, is responsible for huge greenhouse gas and ammonia emissions, and likely contributes to global warming [6,7]. In addition, traditional animal protein consumption has been proven to correlate with risk for cardiovascular disease and cancer [8,9]. Therefore, there is an urgent need for new sources of proteins that are environmentally sustainable and confer high quality to food, maintaining acceptable sensory characteristics. Alternative protein sources for human food include those from plants (legumes, soy, seeds, nuts), algae, fungi, cultured meat, insects and by-products from the food industry [10]. Compared to traditional animal proteins, all these alternatives can offer more sustainable food in terms of environmental impact, representing a potential solution for reducing land and water use in agriculture and, consequently, greenhouse gas emissions and pollution. On the other hand, alternative protein sources are also attractive for their healthy nutritional profile. In addition to their high protein content, they are a good source of essential amino acids and rich in micronutrients such as vitamins and minerals [11]. Such a sustainable alternative source, rich in proteins and other nutrients, can be incorporated into various foods, but often affects their functional properties, such as texture, flavour and shelf life. Nevertheless, in most cases, these alternative protein sources can be chosen or modified to improve the desirable qualities of food products, making them more appealing to consumers [12].

In this scenario, wheat-based staple products, which represent the most consumed in worldwide diets, are suitable to be integrated with innovative ingredients, thus improving the nutritional, quality and sensory profile of the new food [13,14]. Among staple foods, there is pasta whose matrix allows the incorporation/addition of other nutrient-rich ingredients without altering, but likely improving, its well-known nutritional and technological features as documented by the literature [15,16]. Due to their high protein content, there is growing research interest in investigating the addition of legume flours, fruit or vegetable puree/powders and insect powders to a variety of cereal-based foods, including pasta [17,18,19,20,21]. Compared to conventional animal proteins, these alternative protein sources can provide essential amino acids, polyunsaturated fatty acids, minerals, vitamins, antioxidants and fibres required for human nutrition and, at the same time, the opportunity to formulate and commercialise new protein-based food with sustainable materials. In recent decades, the formulations of wheat/semolina dried pasta enriched with agri-food by-products have been the most investigated [22,23]. To the best of our knowledge, only a few authors have investigated those pasta formulations in which wheat flour is partially replaced with cricket powder [15,20,24,25]. In 2013, the Food and Agriculture Organisation (FAO) published a report aimed to promote the consumption of insects as a source of protein for human and animal nutrition due to their nutritional value and its low environmental impact [3] and, more recently, an overview on food safety issues associated with the production and consumption of insects [26]. Recently, the commercialisation of partially defatted powder of *Acheta domesticus* (house cricket), the most commonly known edible insect, has been authorized by European Union (EU) as a novel food ingredient for the preparation of several products including bakery goods, cereal bars, sauces, soups, snacks with corn flour, beer-like beverages, chocolates and also for the formulation of aquaculture feeds [27]. On the other hand, the European Food Safety Authority (EFSA) evaluated the allergenicity risks of consuming insects, concluding that the possible hazards are related to insect feed and processing [28]. To date, no additional hazards in comparison to the more commonly consumed animal products have been reported in the scientific literature. However, the consumption of insects, already common in several countries of East Asia, is still limited in Western countries due to the lack of consumer acceptance. Studies reported that western consumers may be more willing to try insects if they are in non-visible forms, such as cricket powders, than in visible forms (whole insects) [15,21,25]. Cricket powders with a high protein content (42.0–65.5% dry matter) may increase acceptance by western consumers. Moreover, although the hazelnut flour is naturally rich in plant-based proteins, its use in fresh pasta production has not been investigated, whereas hazelnut by-products, such as hazelnut skin, seem to be more interesting for the enrichment of pasta or cookies with high fibre content, antioxidant activity and sensory features [29,30].

When developing novel food products, evaluating their technological properties and predicting their future acceptance are of great importance. These technological characterisations might be investigated through a wide range of standard approaches, such as cooked pasta firmness, cooking loss, water absorption, colour determination and descriptive sensory analysis. Fewer studies have been carried out to evaluate these characteristics in the pasta formulated with alternative protein sources through new approaches such as ^1^H NMR analysis and E-nose. These approaches, in combination with classical technological analysis, have been used in this study to evaluate the quality and sensory properties of traditional egg pasta fortified with hazelnut flour and cricket powder.

## 2. Results and Discussion

### 2.1. Evaluation of Pasta Quality

#### 2.1.1. Standard Cooking Parameters

To evaluate the pasta quality, the optimal cooking times (OCTs) were first determined for the traditional egg pasta samples (control pasta) and those fortified with hazelnut flour (hazelnut pasta) or partially defatted cricket powder (cricket pasta). The OCT significantly decreased (*p*-value < 0.0005) in hazelnut pasta (5.89 ± 0.10 min), while it significantly increased (*p*-value < 0.0005) in cricket pasta (8.67 ± 0.17 min) compared to the control pasta (7.44 ± 0.10 min). Similar to hazelnut pasta, the enrichment of legume flours such as faba bean and split pea also resulted in a reduction in the OCT [31]. After pasta fortification with cricket powder, the increase in OCT could be ascribed to the higher amount of protein and fibre vs. a lower starch content, resulting in a reduction in water diffusion rate [20].

Then, other quality parameters, such as the cooking loss (CL) and water absorption (WA), were measured after the OCTs were determined for each pasta sample and after 20 min, which represents the overcooking time. The CL and WA values in relation to both cooking conditions are reported in Table 1. Despite the increase in the OCT, which means more time in boiling water, cricket pasta did not show any significant difference in the water release of organic substances compared to the control pasta.

The CL, in fact, represents an important parameter to evaluate a quality pasta that tends to lose fewer solids during cooking. It is noteworthy that cricket pasta, whose OCT was the highest, showed a significant reduction in CL when compared to hazelnut pasta. On the contrary, after the pasta fortification with hazelnut flour, despite the decrease in OCT, the CL significantly increased in hazelnut pasta compared to the control pasta. This increase in organic substances in cooking water can be attributed to the formation of a weak gluten matrix/network due to a likely reduction in gluten and concomitant increase in soluble proteins following the addition of hazelnut flour. Moreover, in the overcooking condition (20 min), only hazelnut pasta showed a significant increase in the CL in comparison to the control pasta; notably, the CL of overcooked cricket pasta was significantly lower compared to hazelnut pasta.

Finally, regarding the WA of pasta samples, the decrease was very slight, but significant, after OCT and marked after 20 min only in the cricket pasta vs. control, probably due to its high content of proteins and/or fibres. All effects that were statistically significant were associated with large effect sizes (d > 0.8).

#### 2.1.2. ^1^H NMR Analysis of Cooking Water

**Figure 1:**
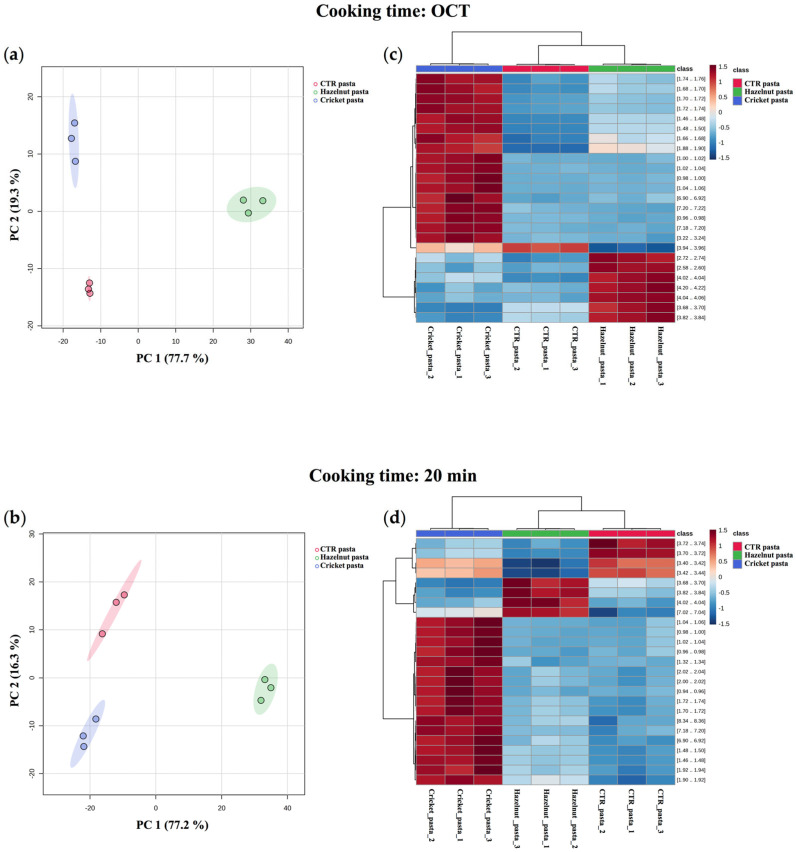
Multivariate statistics applied to the data matrix, prior to assignment, from ^1^H NMR analysis of cooking water. PCA score-plot of cooking water from control (CTR) pasta (red), hazelnut pasta (green) and cricket pasta (blue) samples at (**a**) OCT and (**b**) after 20 min of cooking time. The variance values of PC1 and PC2 are shown in brackets. Each point represents one of three independent replicates for each pasta sample; 95% confidence regions are displayed. Hierarchical Cluster Analysis of cooking water. Dendrogram (top of the panel) combined with a heatmap of the top 25 ANOVA variables (^1^H NMR buckets) following the comparison of cooking water from fortified pasta samples with the control at (**c**) OCT and (**d**) after 20 min of cooking time.

Pasta quality was also assessed by analysing the pasta–water interaction through ^1^H NMR spectroscopy. ^1^H NMR analysis was, in fact, performed on water samples in which the different types of pasta were boiled at the relative predetermined OCT and after 20 min of cooking time. This allowed us to identify differences in the amounts of chemical compounds released by fortified egg pasta samples compared to the control, not only during cooking, but also in the overcooking condition. Principal Components Analysis (PCA) and hierarchical clustering were applied prior to the assignment of ^1^H NMR spectra in order to explore the clustering patterns of pasta samples at both cooking times (Figure 1). PCA analysis revealed a significant separation between the three types of samples, suggesting that the pasta fortification with alternative protein sources significantly affects the pasta matrix mobility by modulating the release of chemical components into the water during the cooking and overcooking processes. Interestingly, cooking water components from hazelnut pasta appeared more different from the control (CTR) pasta compared to the variations observed for the cricket pasta (Figure 1a). The PCA scores plot, in fact, showed that water samples from hazelnut pasta and control pasta were markedly distant from each other along PC1, which accounts for 77.7% of the sample variance. Conversely, the cooking water of cricket pasta was not detached from CTR one along PC1 but was clustered away from it along PC2, which accounts for 19.3% of sample variance. This trend was also maintained in the overcooking condition, where the PC1 and PC2 after 20 min of cooking are responsible for 77.2% and 16.3% of the total sample variance (Figure 1b). Moreover, the results of hierarchical cluster analysis are shown in Figure 1c,d as heatmaps, in which the top 25 ANOVA variables (^1^H NMR features, namely window region of the NMR spectra) correspond to the rows, while the independent replicates are displayed in the columns. For both heatmaps, the horizontal dendrograms show the clustering of the samples based on the NMR features into three distinct groups, thus confirming the pattern observed on the PC2 vs. PC1 plots. The grouping of the NMR features based on their peak areas (vertical dendrograms) shows three clusters for both heatmaps. Among the latter clusters, we can hypothesize that the third cluster of Figure 1c and the second cluster of Figure 1d (clusters numbered from top to bottom) represent variables correlated with PC1 since the NMR peak intensities show high values for the pasta enriched with hazelnut flour and low values for the other two pasta types, the same happens in the case of PC1. Following a similar reasoning for PC2, we can say that for OCT cooking time, the first cluster could represent the variables correlated with PC2. For the cooking time of 20 min, on the other hand, the correlation between PC2 and the clustering of NMR features from the heatmap appears less obvious. Results of this analysis can be viewed as complementary to those obtained from the determination of standard cooking parameters, thus confirming the significant differences in CL, which are visibly fortification-specific and only partially overlap between the two fortified pasta samples.

We then attempted to identify which chemical compounds discriminate the fortified pasta cooking water from the control one and which are specifically present in each of the two different types of fortified pasta. Representative spectra are reported in Figures S1–S3. The most relevant portion in the spectra is the carbohydrate region (3.4–4.5 ppm). Resonances were assigned according to the literature, the library from Chenomx NMR suite 10.0 and 2D TOCSY. A total of 30 metabolites were identified. The chemical shifts of identified metabolites are reported in Table S1 All the water samples were found to contain the same chemical compounds: (i) aliphatic amino acids and organic acids such as malate and succinate (1–3 ppm); (ii) saccharides such as glucose, sucrose, maltose, raffinose (3–5.6 ppm) and (iii) aromatic amino acids and organic acids such as fumarate and formate (5.8–8 ppm).

**Figure 2:**
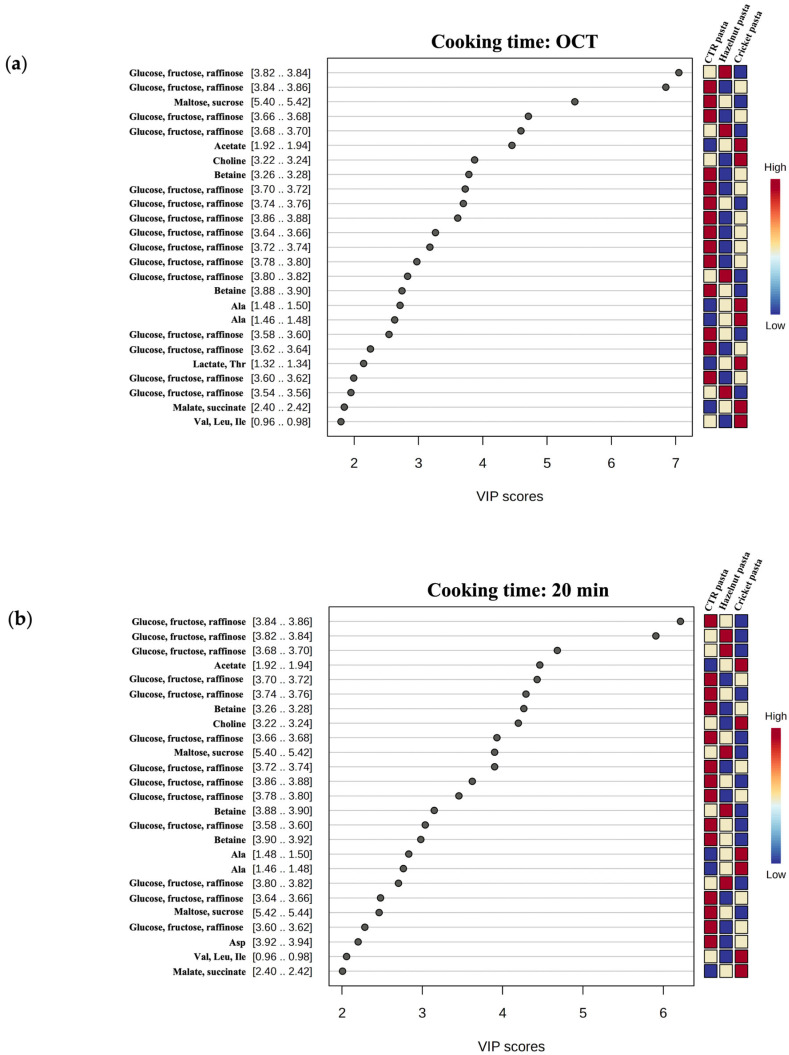
Multivariate statistical analysis of ^1^H NMR data after assignment of cooking water spectrum. VIP values of the 25 most important metabolites in cooking water from control (CTR) pasta, hazelnut pasta and cricket pasta at (**a**) OCT and (**b**) after 20 min of cooking time (*p*-value < 0.05).

After the assignment, Partial Least Squares Discriminant Analysis (PLS-DA) was applied to obtain a better prediction of ^1^H NMR variables (the chemical compounds) and to compare their levels among the three different types of pasta (Figure 2). To demonstrate its validity and robustness, the PLS-DA model selected for NMR data was first validated; in particular, the model for the OCT results contained two latent variables (LVs), and its prediction performance was positively evaluated in cross-validation by Q^2^ = 0.983, R^2^ = 0.993, and accuracy = 1.0. The model for the 20 min results contained three LVs, and showed Q^2^ = 0.959, R^2^ = 0.990, and accuracy = 1.0. The permutation test for the prediction accuracy resulted in a *p*-value of 0.004 and 0.068, for the OCT and 20 min data, respectively.

Moreover, Figure 2a,b shows the Variable Importance in Projection (VIP) values of the 25 most important metabolites and their relative abundance at the OCT and after 20 min of cooking, respectively.

**Figure 3:**
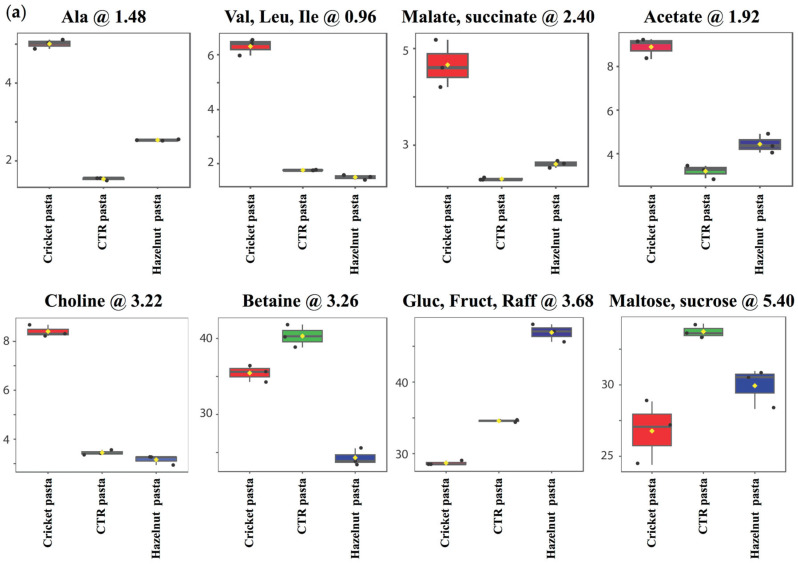

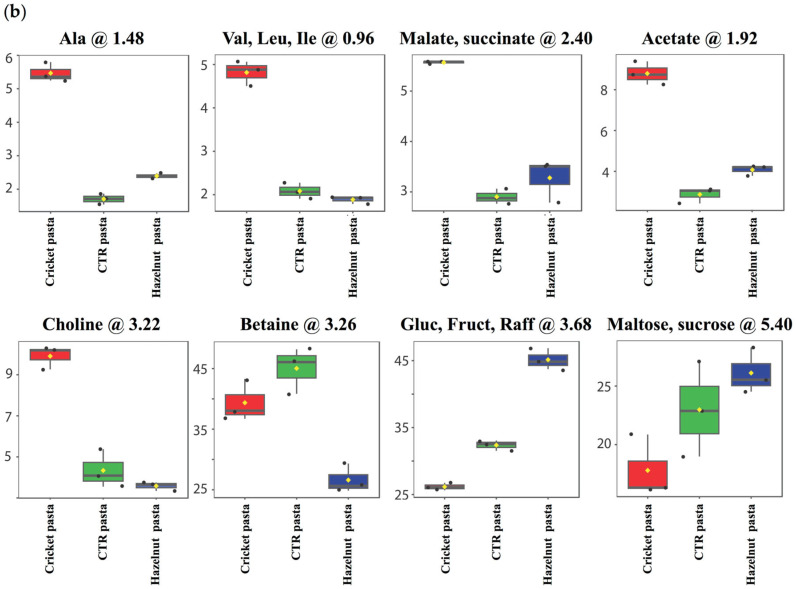
Box plot showing the relative abundance of some metabolites showing significant variation between pasta water samples at (**a**) OCT and (**b**) 20 min of cooking time. In the plot, the boxes represent interquartile ranges, the horizontal line represents the median, the yellow dot represents the mean value. Top and bottom boundaries of the boxes are the 75th and 25th percentiles, while lower and upper whiskers are the 92th and 5th percentiles, respectively. The chemical shift value in ppm for different metabolites is also reported at the top of each plot.

Cricket pasta showed a greater release of aliphatic compounds, such as amino acids (Ala, Val, Leu, Ile) and organic acids (malate, succinate, acetate), and of choline than control (CTR) pasta, regardless of cooking time (Figure 2a,b). On the other hand, hazelnut pasta showed a lower tendency to release these aliphatic compounds or no difference in their levels compared to the control. Moreover, hazelnut pasta also showed a lower release of betaine (Figure 2a). Interestingly, an opposite trend was observed among the three samples regarding the saccharides, whose release significantly decreases for cricket pasta and increases for hazelnut and CTR pasta both at OCT and after 20 min of cooking (Figure 2a,b). To better rationalise these findings, an analysis was carried out to define the relative change in the metabolite levels between the three different samples. Figure 3a,b shows representative box-plots derived from this analysis at OCT and after 20 min of cooking, respectively. The plot reveals the quantitative variation of relative signal integrals for key metabolites altered in cooking water from cricket or hazelnut pasta compared to the control. These metabolites include the aforementioned amino acids, organic acids and saccharides. The differences in the saccharide component release into the water seem to correlate with the observed variations in CL during the cooking or the overcooking processes (Table 1). This suggests that a lower release of these compounds may be related to a more compact matrix of cricket pasta.

#### 2.1.3. Determination of Pasta Colour

The pasta colour, which is the first aspect evaluated by consumers, is significantly affected by the pasta fortification with hazelnut flour or cricket powder. The lightness (L* parameter), in fact, significantly decreased in both fortified uncooked/raw pasta compared to the control pasta (Table 2). Also, other studies reported a decrease in lightness after pasta fortification with edible cricket [20,24].

After cooking, a significant reduction in lightness was confirmed only for cricket pasta when compared with both cooked control and hazelnut pasta (Table 2).

The redness (a * parameter) significantly increased for raw hazelnut pasta compared to the uncooked control, whereas fortification with cricket powder, before cooking, determined a significant reduction in this parameter in comparison to control and, obviously, even more if compared to hazelnut pasta. Although the cooking reduced the redness of all pasta samples compared to their uncooked counterparts, the pasta fortification significantly increased the a * values in both cases compared to the control, and in the cricket pasta respect to the hazelnut one (Table 2). In addition, both fortified pasta samples had yellowness (b * parameter) values significantly lower than those of the control pasta before cooking, and, in this condition, the fortification with cricket powder further decreased the b * values with respect to that with hazelnut flour (Table 2).

Moreover, all cooked pasta samples exhibited lower b * values compared to their uncooked counterparts. Interestingly, after cooking only in the cricket pasta, the lightness and yellowness decreased, while the redness increased compared to the control pasta (Table 2). This observed darkening and higher level of redness after pasta fortification with cricket powder can be ascribed to the pigments contained in the powder, as well as to the Maillard reactions. These may be promoted by the higher protein content of cricket pasta, leading to the higher formation of brown melanoids. The lightness and redness data are consistent with those obtained in other types of pasta fortification, such as with silkworm pupae or legume flours [32]. The mechanistic basis of these colour changes can be linked to both the intrinsic composition of the fortifying ingredients and the chemical reactions occurring during cooking. Cricket powder contains naturally dark pigments, including melanin-like compounds, that contribute to a lower initial lightness (L) and yellowness (b) of the uncooked product [33]. Moreover, its high protein content, combined with the presence of free amino acids and reducing sugars, provides favourable conditions for Maillard reactions during cooking. This non-enzymatic browning process results in the formation of coloured compounds such as melanoidins, which deepen the colour of the product [34]. In the case of hazelnut flour, the lower lightness and higher redness of the uncooked pasta may be attributed to the presence of phenolic compounds and residual pigments from the nut skin [35], while the less intense colour shift upon cooking suggests a lower extent of Maillard browning compared to cricket-enriched pasta.

Given that pasta samples are considered to have different colours when ΔE ≥ 12, the overall colour difference of the cricket pasta (ΔE = 25.69; CI = 20.20, 31.20) or hazelnut pasta (ΔE = 12.10; CI = 4.64, 19.54) with respect to the control can be considered not only statistically significant but also evident, although the difference was more marked for cricket than hazelnut pasta. Interestingly, there was a noticeable, statistically significant colour difference between the cricket pasta and hazelnut one (ΔE = 14.92; CI = 13.44, 16.46).

After cooking, the total colour difference between the hazelnut pasta and control pasta was not statistically significant and only perceptible (ΔE = 4.43; CI = −3.63, 12.50); whereas the colour differences of cricket pasta with control pasta or hazelnut pasta persisted after cooking showing very high and statistically significant ΔE values, equal to 19.22 (CI = 5.37, 33.07) and 15.68 (CI = 14.10, 17.27), respectively.

The total colour differences between cooked and uncooked pasta within the same sample were also evaluated. In particular, the ΔE values between the uncooked and the cooked samples of control or hazelnut pasta were 21.65 (CI = 12.51, 30.80) and 15.61 (CI = 4.89, 26.49), respectively, thus suggesting that both type of pasta during cooking takes on a different colour; the cooked cricket pasta, instead, compared to the same uncooked samples, showed a ΔE value equal to 9.48 (CI= 7.28, 11.98). All statistically significant colour differences had large effects, showing d values in the range 4.0–88.7.

### 2.2. Sensory Evaluation of Cooked Pasta

#### 2.2.1. Sensory Profiling by Descriptive Analysis

Sensory analysis of control and fortified egg pasta samples was performed using key statistical measures, determined coefficients and *p*-values are reported in Table 3. They helped us to determine the significance and the magnitude of differences among the samples. Observing the values of Table 3, it is clear that “Colour” (CO), “Shrimp Flavour” (SF), “Bread Crust” (BC), “Nut” (NU) and “Pitting” (PI) represent the attributes that characterize the products and significantly mark the differences between all the pasta samples.

Table 4 displays the mean value and model coefficients for each combination of pasta sample and descriptors. Each value reported, relating to each single descriptor, based on the model, indicates a significant discriminating positive (blue) or negative (red) effect on the characterisation of the product compared to all the others analysed. The results indicated significant differences in several sensory descriptors among the pasta samples. The control pasta typically showed neutral characteristics, with expected notes of “Egg” (EG) and a mild “Cooked Odour” (CKO). Moreover, significant differences were observed in tactile attributes, with the control pasta presenting a more homogeneous texture.

In contrast, the cricket pasta displayed a marked increase in “Vegetal Dried” (VD) and “Shrimp flavour” (SF) descriptors. These results are in line with previous findings by Grossman et al. who identified 30 odour-active compounds, including alcohols, acids, aldehydes, ketones, and heterocyclic compounds such as pyrazines, related to both cricket and shrimp [36]. The perception of SF in our study is also consistent with Umebara et al. who included “shrimp-like odour” among the sensory descriptors for edible cricket powder [37].

The hazelnut pasta exhibited distinct flavour attributes, including pronounced nutty (NU) notes accompanied by increased saltiness (SA) and bitterness (BI). Hazelnut pasta also displayed a marked astringency (AS), which can be explained by the presence of tannins, which are abundant in hazelnuts and are recognised for their ability to induce astringency [38]. Both enriched pasta samples presented a rougher surface texture (SR) and increased pitting (PI) compared to the control. The cricket pasta exhibited a higher colour intensity (CO). An increase in colour intensity, specifically a darkening of the product following the use of cricket flour, has been previously reported in the literature [39,40], potentially related to the presence of natural pigments derived from the insects. Notably, the descriptors NU and CKO for hazelnut pasta and VD and SF for cricked pasta played a crucial role in differentiating between the enriched and control pasta.

The intensity of these descriptors was significantly higher in the enriched types of pasta, reflecting the solid aromatic influences of the added ingredients. Furthermore, “Homogeneous Texture” (HT) served as a critical descriptor in evaluating texture changes. The cricket pasta showed a less uniform consistency, reflecting the influence of cricket flour on the pasta texture.

#### 2.2.2. Sensory Analysis with Electronic Nose

The E-nose, functioning as a detection instrument, can identify simple and complex aromas through an array of non-specific sensors and a well-suited pattern recognition system. The aromatic fingerprint was, therefore, also evaluated using the QMB E-nose. When molecules bind to the coated layer on the surface of the E-nose QMB, it causes a change in their oscillation frequency. This change is proportional to the amount of absorbed mass, leading to a measurable shift in the electrical signal. QMBs are made from thin quartz discs, usually coated with different materials, which provide each sensor a different affinity for different volatile components [41]. Therefore, the ability of an E-nose system to distinguish between samples depends on its capability to generate a measurable response to different aroma components and to differentiate between aromas with varying compositions.

**Figure 4:**
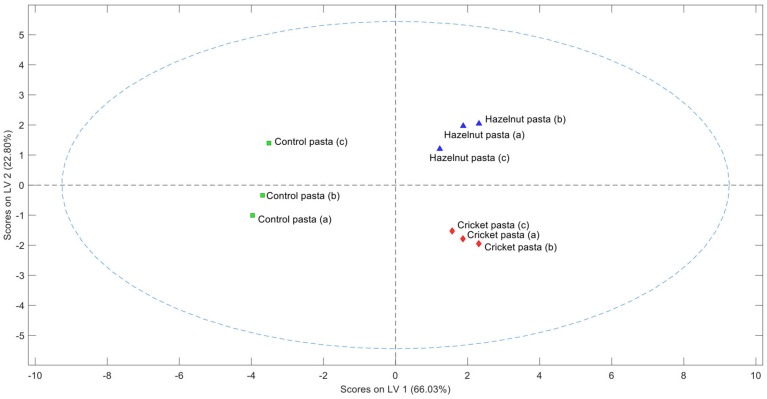
Score plot (LV1 vs. LV2) of the PLS-DA built with autoscaled E-nose data. Each letter in parentheses represents one of three independent replicates for each pasta sample.

After autoscaling E-nose data, PLS-DA analysis was applied and the relative confusion matrix is reported in Table S2. The PLS-DA model built with two LVs was selected for the sensory data, as it showed satisfactory cross-validation performance, with Q^2^ = 0.506, R^2^ = 0.898, and classification accuracy of 1.0. The PLS-DA model describes about 88.83% of the variability on the first two LVs (LV1 66.03% and LV2 22.80%) (Figure 4).

PLS-DA score plot analysis is usually based on the assumption that the distance between points indicates the similarity among samples. Consequently, clusters observed in the score plots are construed as groups of comparable samples. The plot revealed the presence of three distinct clusters. The first cluster included control pasta, the second the cricket pasta, while the third comprised the hazelnut pasta. This suggests that there are notable aromatic differences among various pasta samples, as already highlighted by the sensory analysis. The results indicate that the model perfectly discriminates the different samples, suggesting high differences between the different samples.

## 3. Materials and Methods

### 3.1. Raw Materials

Commercial raw materials were utilised for egg pasta production and their compositions per 100 g, available on the label, are reported below:

- soft wheat flour “00” (Molino Profili, Viterbo, Italy): 73 g of total carbohydrates, 11.5 g of proteins, 2.0 g of total fat and 2.5 g of total dietary fibre;
- durum wheat semolina (Molino Profili, Viterbo, Italy): 72 g of total carbohydrates, 11.5 g of proteins, 1.0 g of total fat and 2.5 g of total dietary fibre;
- hazelnut flour of the Tonda Gentile Romana (Agricola Pini, Viterbo, Italy): 8.9 g of total carbohydrates, 17.9 g of proteins, 64.8 g of total fat and 4.0 g of total dietary fibre;
- partially defatted cricket (*Acheta domesticus*) powder (Small Giants, Milan, Italy): 3.2 g of total carbohydrates, 77.2 g of proteins, 11.6 g of total fat and 8.0 g of total dietary fibre;
- eggs were purchased by Azienda Agricola Bacocco (Viterbo, Italy).

To ensure consistency among experimental replicates, all raw materials were obtained from single production batches.

### 3.2. Pasta Manufacturing

“Control egg pasta” was prepared using a mixture of soft wheat flour “00” and durum wheat semolina in the ratio of 1:1 (*w/w*), and then adding the egg in the ratio of 1:2 (*w/w*) to the total weight of flours.

Fortified egg pasta samples were prepared by replacing 15% (*w/w*) of total traditional flour with hazelnut one or partially defatted cricket powder (*Acheta domesticus*). In detail, 42.5 g of soft wheat flour “00”, 42.5 g of durum wheat semolina, 50 g of egg added with 15 g of hazelnut flour or 15 g of cricket powder were mixed and kneaded by hand. During pasta preparation, the temperature was maintained at 18–22 °C and the relative humidity around 50–60%. The dough was thinned and rolled out to a thickness of about 0.20 cm and cut into 0.65 cm wide long strips by using a manual pasta machine (Marcato ATLAS 150, Padova, Italy). The final shape was obtained by cutting the long strips into 5 cm length short strips. These strips were dried for 8 h at 55 °C using a vertical food dryer. Egg pasta samples were stored in sealed containers at room temperature before the analyses. These were performed on at least three independent replicates corresponding to egg pasta samples (control and fortified ones) prepared and cooked at different times.

### 3.3. Standard Parameters of Pasta Quality

*Cooking parameters.* To ensure experimental reproducibility, distilled water without any added salt was used in the cooking tests, and the water-to-pasta ratio was maintained constant throughout. Specifically, five grams of pasta samples were cooked in 150 mL of boiling distilled water. Pasta was added to the water once it started boiling. The optimal cooking time (OCT) and cooking loss (CL) were determined based on the AACC methods 44-15A and 66–50 immediately after the cooking process [42]. Briefly, after draining and washing the cooked pasta, the water used for cooking and rinsing was gathered in a pre-weighted container and evaporated in an oven at 105 °C. CL was calculated as the percentage of the residue.

After weighing the cooked pasta, the water absorption (WA) was calculated as the weight percentage compared to that of the uncooked pasta samples.

*Determination of colour*. The colour of egg pasta samples was measured using a Minolta colourimeter (Minolta C2500; Konica Minolta, Ramsey, NJ, USA) according to the CIELAB system [43]. The chromaticity values L* (lightness), a* (redness), and b* (yellowness) were recorded for at least ten different samples for each pasta. The colour difference (ΔE) between pasta samples was calculated as previously reported [21].

### 3.4. ^1^NMR Spectroscopy of Cooking Water

Each pasta sample (3 g) was cooked in 50 mL of distilled water for relative OCT for 20 min. The water cooking was carefully withdrawn, centrifuged to remove suspended particles, and concentrated in a vacuum centrifuge immediately before the NMR analysis. Then, 500 μL of each sample in phosphate buffer suitable for ^1^H NMR experiments was placed into a 5 mm NMR tube for acquisition of ^1^H NMR spectra as previously described [44].

Samples were locked on the deuterium signal of D_2_O and the magnetic field homogeneity was optimised for each sample. The spectral width was 6410 Hz, and 32 k data points were collected using the standard Bruker NOESY pulse sequence (*noesypr1D*) and collected after a 100 ms mixing period. The water suppression was achieved using continuous wave irradiation. All spectra were recorded using 64 transients, a relaxation delay of 2 s, an acquisition time of 4 s. Processing (phase and spectra baseline correction) was performed with 1D NMR Manager 12.0 software (ACD/Labs, Toronto, ON, Canada) and the spectra were referenced to the singlet signal of sodium 3-trimethylsilyl [2,2,3,3-d4] propionate as previously reported [44].

For compound identification, ^1^H-^1^H total correlation spectroscopy (TOCSY) 2D experiments were acquired with a spectral width of 10 ppm in both dimensions and a mixing time of 70 ms. All NMR experiments were performed on three independent replicates. The spectral region corresponding to the solvent (water) δ 4.54–5.22 was excluded, and the remaining regions were divided into 0.02 ppm bins. Thus, NMR spectra of cooking water samples were represented by 416 bins, integrated and transformed into a comma-delimited form (CSV). The data matrix of bucket area of nine samples (rows) × 416 chemical shifts (columns) was analysed by using Metaboanalyst 6.0 software (http://www.metaboanalyst.ca, Wishart Research Group, Edmonton, AB, Canada). Before the assignment of NMR spectra, Principal Component Analysis (PCA) and hierarchical cluster analysis were performed after normalisation by the sum of the bucket area. Metabolites, identified as previously described [44], are reported in Figures S1–S3 and Table S1. After the assignment, Partial Least Squares Discriminant Analysis (PLS-DA) was applied to identify the most relevant ^1^H NMR variables (the chemical compounds) using the VIP (variable importance in projection) scores > 1 and *p*-value < 0.05. To this aim, the PLS-DA model was optimised and internally validated by 5-fold cross-validation; the maximum number of latent variables (LVs) was set to three, and the number of LVs was selected according to the highest Q^2^ value obtained in cross-validation. Model performance was assessed by estimating Q^2^, R^2^, and prediction accuracy statistics in cross-validation. Undesirable features, like model overfitting, random behaviour, and low prediction power, were assessed through permutation tests (with 1000 permutations) of the prediction accuracy statistics.

### 3.5. Sensory Profile

*Descriptive sensory analysis*. The sensory test was performed by a panel of eight expert sensory judges (three males and five females, aged between 26 and 39) recruited from the staff of Tuscia University (Viterbo, Italy) and in a sensory laboratory as previously described [45]. Assessors agreed upon the descriptor definitions of the products’ sensory profiles (Table S3), scale anchors, and evaluation techniques during training (Table S4). The judges were given three single units of each of the three samples. The pasta stripes had a length of about 3 ± 0.2 cm and a width of 0.7 ± 0.1 cm, with a thickness of about 0.2 ± 0.0 cm, and were coded with three random numbers.

*Electronic nose measurement*. A quartz microbalance (QMB)-based electronic nose (E-nose) was employed to further analyse the aromatic profile. This E-nose consists of an array of 12 QMBs designed and functionalized as previously described [41]. The instrument control and data acquisition are handled by custom software developed in MATLAB (MathWorks^®^, Natick, MA, USA) [41]. The measurement protocol involves incubating 3 g of cooked pasta in sealed vials at 30 ± 1 °C for 20 min. The equilibrated headspace was then extracted for 60 s using filtered air and introduced into the E-nose sensor chamber. After each measurement, the system was cleaned for 180 s. The sensor signals were calculated by measuring the resonant frequency shift between the sensors exposed to pure air and the sample. Three separate vials were analysed for each sample.

### 3.6. Statistical Analysis

Results are reported as mean ± standard deviation (SD) and 95%-confidence interval (CI) from at least three independent replicates. Data from quality parameter analysis were statistically validated using the two-sample Student’s *t*-test. The significance of the difference threshold was set at a *p*-value < 0.05. To measure the magnitude of the effects for CL and WA data, the absolute value of Cohen’s d was estimated as the absolute value of the difference between the means being compared divided by the corresponding pooled standard deviation. For ΔE data, the statistical significance was tested by reporting the associated 95%-confidence intervals (equivalent to one-sample Student’s *t*-test), while the Cohen’s d values were calculated as the ratio between the mean and the standard deviation.

Data collected from the sensory evaluation were also processed using the XLSTAT software (Lumivero, Denver, CO, USA) according to the *product characterisation test* [45]. This approach also helps characterise the sensory profile through the most essential product’s sensory descriptors. For each descriptor, an ANOVA model is applied to check whether the scores assigned by the judges are significantly different, thus considering not only the “product” effect but also the “judge” effect for each evaluation. The data processing result returns various coefficients of the model chosen for each combination product-descriptor (Table S5). E-nose numerical data were first autoscaled and then analysed with PLS-DA using Venetian-blind as a validation method (with a blind thickness = 1). The statistical significance of the PLS-DA model was assessed through a permutation test (*n* = 1000 permutations) using the prediction accuracy during training as a performance metric. The class labels were randomly permuted to generate a distribution of accuracies from random models, and the *p*-value was calculated as the proportion of permuted models yielding an accuracy equal to or greater than the original model.

Multivariate analysis was performed by using MATLAB R2013a and PLS Toolbox (Eigenvector Research, Inc., Manson, WA, USA).

## 4. Conclusions

This study aimed to implement and enhance the knowledge on the changes in quality and sensory features due to the addition of nutrient-rich ingredients in food such as pasta. This highlights the necessity to expand the variety of wheat-derived products enriched with sustainable ingredients.

From a quality point of view, pasta samples fortified with the two alternative protein sources showed different technological characteristics compared to their conventional counterpart. Interestingly, the cricket pasta, with the highest OCT, showed a significant reduction in CL compared to hazelnut pasta, suggesting that pasta fortification with cricket powder or hazelnut flour differentially affects the matrix mobility and the gluten network formation by modulating the release of chemical components into the water during the cooking and overcooking processes. Notably, these technological aspects were consistent with the molecular characterisation through ^1^H NMR analysis of metabolites released during pasta cooking. Cricket pasta showed a greater release of aliphatic compounds, such as amino acids (Ala, Val, Leu, Ile) and organic acids (malate, succinate, acetate), and a lower release of saccharides (maltose, sucrose) than control pasta, regardless of cooking time, while hazelnut pasta showed a lower tendency to release aliphatic compounds and a higher release of saccharides compared to the control in both tested cooking conditions. The release of different metabolites into the cooking water correlates with the observed variations in CL between cricket and hazelnut pasta, thus affecting the pasta matrix mobility that could be more compact for the cricket pasta compared to the hazelnut one.

The pasta fortification with either hazelnut flour or cricket powder significantly affected the colour of uncooked egg pasta and probably consumer acceptability. The significant colour changes of uncooked fortified pasta compared to control can be attributed to the different pigments contained in the respective protein sources, whereas the persistence of colour differences only for cooked cricket pasta may be linked to its biochemical composition, which favours the Maillard reactions during cooking.

The sensory evaluation demonstrated that cricket and hazelnut flours significantly alter the sensory profile of egg pasta, particularly in terms of aroma and texture. Notably, aromatic differences among pasta samples were also highlighted by the sensory analysis with the E-nose. This technology has emerged as a valuable tool in the study and quality assessment of pasta, particularly in evaluating the volatile compounds that contribute to its aroma.

In conclusion, our results underscore the complexity of striking a balance between nutritional enhancement, technological quality, and sensory appeal in food product development. Although these enrichments contribute to the nutritional value of pasta, they also impose changes that may influence consumer acceptance, depending on individual preferences in terms of taste and texture.

Moreover, this study demonstrated the potential of ^1^H NMR spectroscopy and E-nose technology in pasta analysis and food composition/quality assessment, offering a rapid, cost-effective, and reliable method for evaluating the molecular profile and sensory features of various products, respectively. With the ongoing advancement of technology, new methodologies are expected to become an increasingly integral part of the food industry, helping producers ensure that their products always achieve high standards of quality and flavour. In addition, the use of hazelnut flour and cricket powder as fortifying agents is compatible with existing pasta production processes, which supports the industrial scalability of the proposed formulations. These ingredients could be introduced in normal productions with minimal adjustments, enabling efficient upscaling. From a commercial standpoint, both ingredients offer significant marketing opportunities: cricket powder, for its high protein content and environmental sustainability, and hazelnut flour, for its appeal as a natural, plant-based by-product, positioning the fortified pasta as a value-added product within health- and sustainability-conscious food sectors. Therefore, the practical application of these findings offers concrete opportunities for innovation in the development of functional food.

## Figures and Tables

**Table 1:** Cooking quality parameters of control and fortified egg pasta samples.

Sample	Cooking Loss (CL) (%)			Water Absorption (WA) %		
	mean ± SD	CI	d	mean ± SD	CI	d
Cooking time: OCT						
Control pasta	7.03 ± 0.36	6.14, 7.91	3.18	233.91 ± 1.77	229.50, 238.32	0.03
Hazelnut pasta	8.89 ± 0.62 *	7.35, 10.43	3.68	220.87 ± 12.5	189.75, 251.98	1.46
Cricket pasta	7.10 ± 0.50 ^#^	5.88, 8.34	0.19	221.12 ± 3.85 *	211.56, 230.68	4.27
Cooking time: 20 min						
Control pasta	3.84 ± 0.78	2.60, 5.08	1.80	323.09 ± 4.63	311.58, 334.61	3.20
Hazelnut pasta	5.95 ± 0.71 *	4.82, 7.08	2.84	317.65 ± 9.53	293.98, 341.32	0.73
Cricket pasta	4.64 ± 0.75 ^#^	3.45, 5.83	1.05	286.02 ± 10.25 **^,#^	260.55, 311.48	4.66

Control pasta: egg pasta formulated with 50% soft wheat flour “00” and 50% durum wheat semolina. Hazelnut pasta or cricket pasta: 42.5:42.5:15 (soft wheat flour “00”):(durum wheat semolina):(hazelnut flour/cricket powder, respectively). Data are means ± standard deviation (SD), 95%-confidence interval (CI) and Cohen’s d absolute value (d) of three (*n* = 3) or four (4) independent replicates (with at least two repeats for each replicate). * *p*-value < 0.05, ** *p*-value < 0.005, fortified pasta samples vs. control. **^#^** *p*-value < 0.05, cricket-fortified pasta vs. hazelnut-fortified pasta. Cohen’s d absolute values represent effects comparing, respectively, in order of entry hazelnut pasta vs. cricket pasta, hazelnut pasta vs. control, and cricket pasta vs. control.

**Table 2:** Colour profile analysis of uncooked and cooked control and fortified egg pasta samples.

Sample	Uncooked/Cooked	L * (Lightness)	a * (Redness)	b * (Yellowness)
Control pasta	Uncooked	70.46 ± 0.66	5.23 ± 0.29	41.79 ± 2.08
	Cooked	65.49 ± 1.36	−1.80 ± 0.05	22.54 ± 0.66
Hazelnut pasta	Uncooked	60.61 ± 1.77 **	6.91 ± 0.31 **	34.98 ± 0.39 *
	Cooked	62.54 ± 0.44	0.92 ± 0.28 *	21.07 ± 1.90
Cricket pasta	Uncooked	54.68 ± 0.68 ***^,#^	4.34 ± 0.14 *^,###^	21.53 ± 0.35 ***^,###^
	Cooked	48.60 ± 0.84 **^,##^	2.15 ± 0.16 **^,#^	14.41 ± 1.18 *

Data are means ± standard deviation of at least two independent replicates (each performed on 10 pasta samples). * *p*-value < 0.05, ** *p*-value < 0.005, *** *p*-value < 0.0005 fortified pasta samples vs. control. **^#^** *p*-value < 0.05, **^##^** *p*-value < 0.005, **^###^** *p*-value < 0.0005 cricket-fortified pasta vs. hazelnut-fortified pasta.

**Table 3:** Various coefficients of the model chosen for each pasta sample-descriptor combination and *p*-value.

	Control Pasta		Hazelnut Pasta		Cricket Pasta	
Descriptors	Coefficient	*p*-Value	Coefficient	*p*-Value	Coefficient	*p*-Value
Colour (CO)	−1.389	<0.0001	−0.222	0.018	1.611	<0.0001
Pitting (PI)	−2.167	<0.0001	0.833	0.002	1.333	<0.0001
Surface Roughness (SR)	−1.222	0.000	0.444	0.049	0.778	0.003
Egg (EG)	0.500	0.046	0.500	0.046	−1.000	0.001
Nut (NU)	−0.889	0.000	1.778	<0.0001	−0.889	0.000
Cooked Odour (CKO)	0.556	0.005	−0.111	0.496	−0.444	0.018
Bread Crust (BC)	−1.000	<0.0001	1.667	<0.0001	−0.667	0.000
Vegetal Dried (VD)	−0.944	0.001	−0.278	0.173	1.222	<0.0001
Salty (SA)	−1.000	0.002	0.667	0.021	0.333	0.201
Bitter (BI)	−0.778	0.001	1.222	<0.0001	−0.444	0.033
Aftertaste (AT)	−0.833	0.000	0.333	0.065	0.500	0.011
Taste Intensity (TI)	−0.444	0.041	0.389	0.067	0.056	0.775
Astringent (AS)	−1.111	<0.0001	0.889	0.000	0.222	0.188
Homogeneous Texture (HT)	−1.500	<0.0001	−0.500	0.033	−1.000	0.001
Shrimp Flavour (SF)	−0.889	<0.0001	−0.889	<0.0001	1.778	<0.0001

**Table 4:** Adjusted averages for each pasta sample-descriptor combination. Colours indicate a significant positive effect (blue) and a significant negative effect (red). Colour (CO), Pitting (PI), Surface Roughness (SR), Egg (EG), Nut (NU), Cooked Odour (CKO), Bread Crust (BC), Vegetal Dried (VD), Salty (SA), Bitter (BI), Aftertaste (AT), Taste Intensity (TI), Astringent (AS), Homogeneous Texture (HT), Shrimp Flavour (SF).

Sample	AT	PI	SR	SA	AS	TI	CO	VD	SF	BI	BC	NU	EG	CKO	HT
Control pasta	2.500	4.167	3.667	2.000	1.500	2.333	5.000	2.500	2.667	0.833	0.500	0.000	2.667	2.333	1.833
Hazelnut pasta	2.333	3.667	3.333	2.333	2.167	2.667	3.167	1.000	0.000	2.500	2.833	2.667	4.167	2.667	2.333
Cricket pasta	1.167	0.667	1.667	0.667	0.167	1.833	2.000	0.333	0.000	0.500	0.167	0.000	4.167	3.333	4.333

## Data Availability

The original contributions presented in the study are included in the article; further inquiries can be directed to the corresponding authors.

## Supplementary Materials

The following supporting information can be downloaded at https://www.mdpi.com/article/10.3390/molecules30163438/s1, Figure S1: 400 Mhz 1D ^1^H NMR spectral traces of cooking water from control pasta; Figure S2: 400 Mhz 1D ^1^H NMR spectral traces of cooking water from hazelnut pasta; Figure S3: 400 Mhz 1D ^1^H NMR spectral traces of cooking water from cricket pasta; Table S1: ^1^H NMR signal assignments; Table S2: Confusion matrix of the PLS-DA built autoscaled E-nose data. Err, total error; FNR, false negative ratio; FPR, false positive ratio; F1, F1-score; N, number of classes; P, precision = total positive (TP)/total positive + false positive; TNR, true negative ratio; TPR, true positive ratio; Table S3: Sensory profile sheet; Table S4: Defining the sensory attributes of the profile card; Table S5: Sensory descriptors ranked by their level of discrimination on products, along with their corresponding *p*-values.
