# Supplementary material for: NMR-Based Metabolomic and QMB-Based E-Nose Approaches to Evaluate the Quality and Sensory Features of Pasta Fortified with Alternative Protein Sources
Source: Molecules. 2025 Aug 20;30(16):3438. doi: 10.3390/molecules30163438 (PMC12388352; doi:10.3390/molecules30163438)
Supplement: Supplementary file 1 [file molecules-30-03438-s001.zip › molecules-3773756-supplementary.pdf]

Figure S1

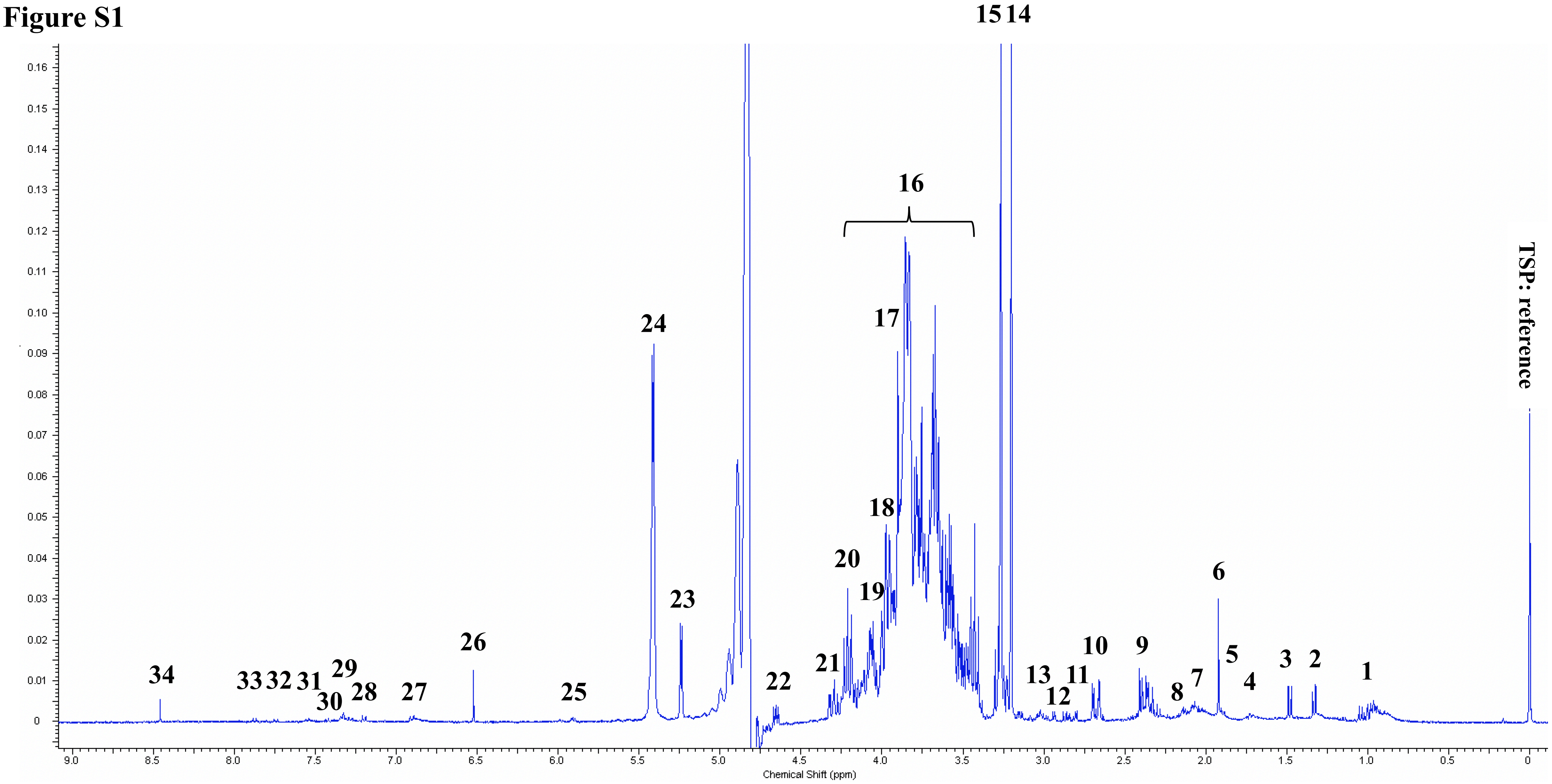

Figure S1: 400 Mhz 1D ^{1}H NMR spectral traces of cooking water from control pasta. 1: Val/Leu/Ile; 2: Lactate/Thr; 3: Ala; 4: Leu/Arg/Lys; 5: GABA/Lys/Arg; 6: Acetate; 7: Pro; 8: GABA; 9: Malate/Succinate; 10: Malate; 11: Asp; 12: Asn/Asp; 13: GABA/Lys/Phe; 14: Choline; 15: Betaine; 16: Glucose/fructose/raffinose; 17: Betaine; 18: Asp; 19: Glycolate; 20: Thr; 21: Malate; 22: Glucose; 23: α-Glucose; 24: Maltose/sucrose; 25: Uridine; 26: Fumarate; 27: Tyr/Tyramine; 28: Trp/Tyr; 29: Trp; 30: Phe; 31: Uracil/Trp; 32: Trp; 33: Uridine; 34: Formate.

Figure S2

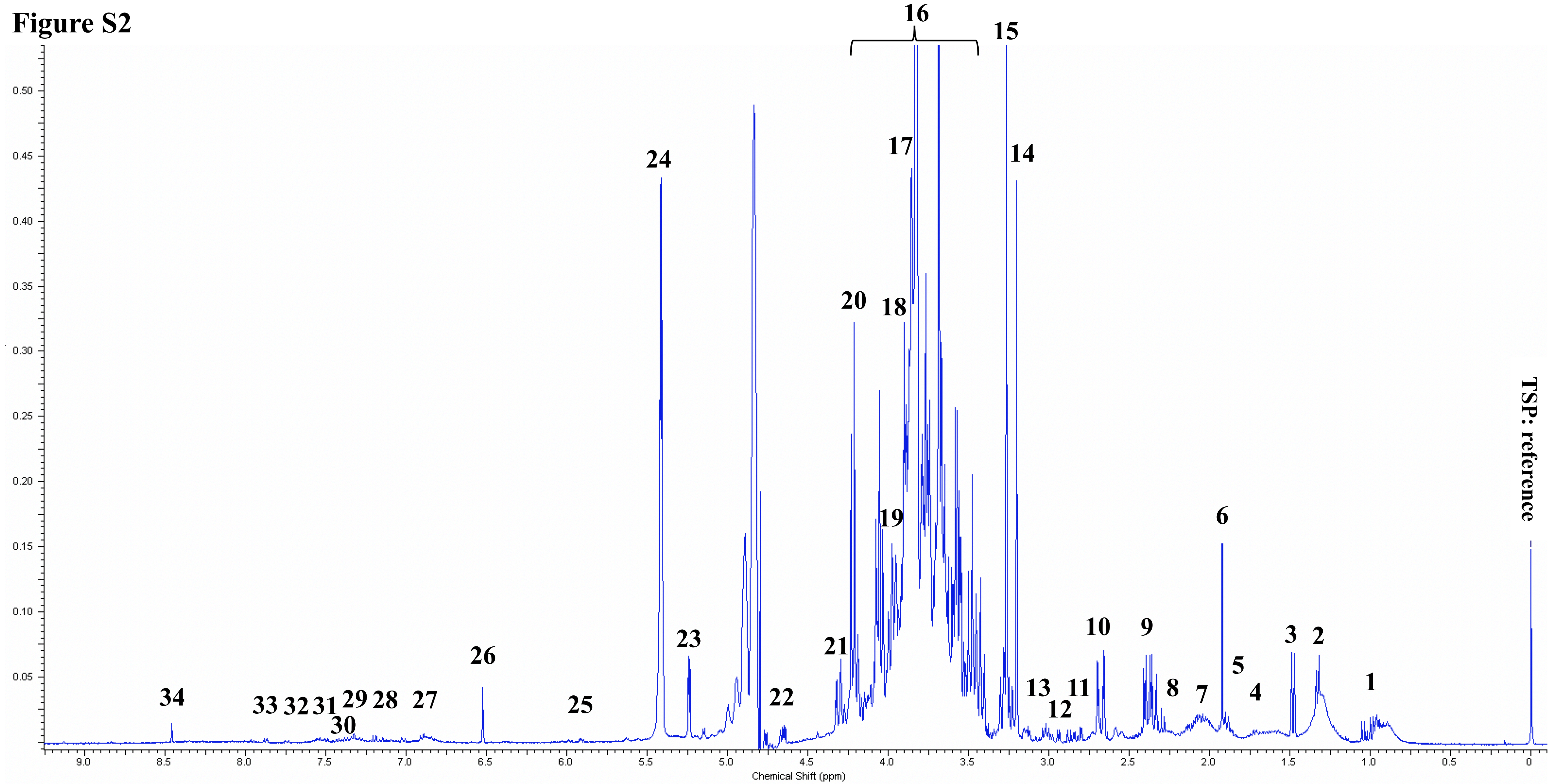

Figure S2: 400 Mhz 1D ^{1}H NMR spectral traces of cooking water from hazelnut pasta. 1: Val/Leu/Ile; 2: Lactate/Thr; 3: Ala; 4: Leu/Arg/Lys; 5: GABA/Lys/Arg; 6: Acetate; 7: Pro; 8: GABA; 9: Malate/Succinate; 10: Malate; 11: Asp; 12: Asn/Asp; 13: GABA/Lys/Phe; 14: Choline; 15: Betaine; 16: Glucose/fructose/raffinose; 17: Betaine; 18: Asp; 19: Glycolate; 20: Thr; 21: Malate; 22: Glucose; 23: α-Glucose; 24: Maltose/sucrose; 25: Uridine; 26: Fumarate; 27: Tyr/Tyramine; 28: Trp/Tyr; 29: Trp; 30: Phe; 31: Uracil/Trp; 32: Trp; 33: Uridine; 34: Formate.

Figure S3

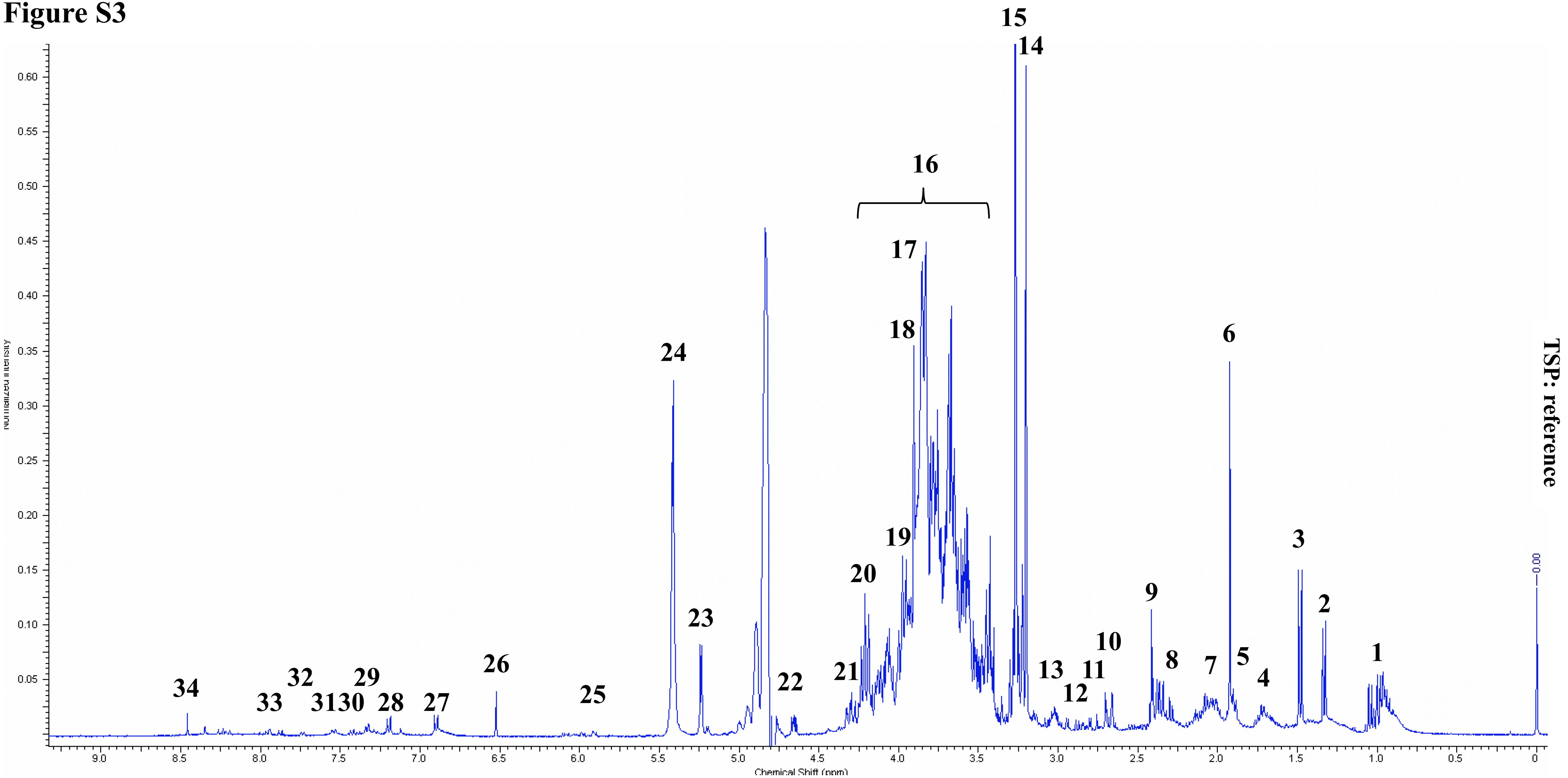

Figure S3: 400 Mhz 1D ^{1}H NMR spectral traces of cooking water from cricket pasta. 1: Val/Leu/Ile; 2: Lactate/Thr; 3: Ala; 4: Leu/Arg/Lys; 5: GABA/Lys/Arg; 6: Acetate; 7: Pro; 8: GABA; 9: Malate/Succinate; 10: Malate; 11: Asp; 12: Asn/Asp; 13: GABA/Lys/Phe; 14: Choline; 15: Betaine; 16: Glucose/fructose/raffinose; 17: Betaine; 18: Asp; 19: Glycolate; 20: Thr; 21: Malate; 22: Glucose; 23: α-Glucose; 24: Maltose/sucrose; 25: Uridine; 26: Fumarate; 27: Tyr/Tyramine; 28: Trp/Tyr; 29: Trp; 30: Phe; 31: Uracil/Trp; 32: Trp; 33: Uridine; 34: Formate.

Table S1. ^{1}H NMR signal assignments.

Metabolite	Chemical shift [ppm]
**Acetate**	1.92 (s)
**Alanine**	1.48 (d)
**Arginine**	1.67 (m)<br>1.90 (m)
**Aspartate**	2.68 (m)<br>2.83 (m)<br>3.93 (m)
**Asparagine**	2.86 (m)<br>2.97 (m)<br>4.01 (m)
**Betaine**	3.27 (s)<br>3.90 (s)
**Choline**	3.20 (s)<br>3.60 (m)<br>4.15 (m)
**Formate**	8.46 (s)
**Fumarate**	6.52 (s)
**Fructose**	3.69 (m)<br>3.90 (m)<br>4.12 (m)
**Glycolate**	3.95 (s)
**Glucose**	3.25 (m)<br>4.65 (d)<br>5.24 (d)
**Isoleucine**	0.95 (m)<br>3.68 (d)
**Lactate**	1.33 (d)<br>4.12 (m)
**Leucine**	0.97 (m)<br>1.71 (m)
**Lysine**	1.72 (m)<br>1.90 (m)<br>3.02 (m)
**Malate**	2.35 (m)<br>2.67 (dd)<br>4.31 (dd)
**Maltose**	5.44 (d)
**Phenylalanine**	3.14 (m)<br>7.37 (m)
**Proline**	2.08 (m)<br>2.35 (m)
**Raffinose**	4.21 (s)<br>5.00 (d)
**Succinate**	2.42 (s)
**Sucrose**	5.41 (d)
**Threonine**	1.33 (d)

	3.60 (d)<br>4.21 (m)
**Tryptophan**	7.29 (m)<br>7.33 (s)<br>7.55 (d)<br>7.74 (d)
**Tyrosine**	6.90 (d)<br>7.20 (d)
**Uracil**	7.54 (d)
**Uridine**	5.91 (m)<br>7.87 (d)
**Valine**	1.04 (d)<br>0.99 (d)
**4-aminobutyrate (GABA)**	1.91 (m)<br>2.30 (t)<br>3.02 (t)

Table S2. Confusion matrix of the PLS-DA built with autoscaled e-nose data. Err, total error; FNR, false negative ratio; FPR, false positive ratio; F1, F1-score; N, number of classes; P, precision = total positive (TP)/total positive + false positive; TNR, true negative ratio; TPR, true positive ratio; and R_2.

MODEL RESULTS – Calibration

**Class**	**TPR**	**FPR**	**TNR**	**FNR**	**N**	**Err**	**P**	**F1**	**R_2 Cal**
Cricket pasta	1.00000	0.00000	1.00000	0.00000	3	0.00000	1.00000	1.00000	0.893
CTR pasta	1.00000	0.00000	1.00000	0.00000	3	0.00000	1.00000	1.00000	0.984
Hazelnut pasta	1.00000	0.00000	1.00000	0.00000	3	0.00000	1.00000	1.00000	0.852

**Predicted as**	**Actual: Cricket pasta**	**Actual: CTR pasta**	**Actual: Hazelnut pasta**	
Cricket pasta	3	0	0	
CTR pasta	0	3	0	
Hazelnut pasta	0	0	3	
Unassigned	0	0	0	

MODEL RESULTS – Cross validation

**Class**	**TPR**	**FPR**	**TNR**	**FNR**	**N**	**Err**	**P**	**F1**	**R_2 CV**
Cricket pasta	1.00000	0.00000	1.00000	0.00000	3	0.00000	1.00000	1.00000	0.753
CTR pasta	1.00000	0.00000	1.00000	0.00000	3	0.00000	1.00000	1.00000	0.962
Hazelnut pasta	1.00000	0.00000	1.00000	0.00000	3	0.00000	1.00000	1.00000	0.680

**Predicted as**	**Actual: Cricket pasta**	**Actual: CTR pasta**	**Actual: Hazelnut pasta**	
Cricket pasta	3	0	0	
CTR pasta	0	3	0	
Hazelnut pasta	0	0	3	
Unassigned	0	0	0	

Table S3. Sensory profile sheet.

Pasta sensory profile	**Date:**
Taste the samples in order and rate each attribute for intensity on a scale of 0 to 5. ***Re-tasting the samples is permitted to align the scores.***	
**Assessor name:**	
**Code samples:**	
**ATTRIBUTES**	
COLOR (CO)	
PITTING (PI)	
SURFACE ROUGHNESS (RG)	
EGG (EG)	
NUT (NU)	
COOKED ODOR (CKO)	
BREAD CRUST (BC)	
VEGETAL DRIED (VD)	
SALTY (SA)	
BITTER (BI)	
AFTERTASTE (AT)	
ASTRINGENT (AS)	
HOMOGENEOUS TEXTURE (HT)	
SHRIMP FLAVOUR (SF)	
TASTE INTENSITY (TI)	

Table S4. Defining the sensory attributes of the profile card.

ATTRIBUTE	DEFINITION
COLOR (CO)	Chromatic intensity
PITTING (PI)	Presence of small white or dark dots on the surface of the pasta
SURFACE ROUGHNESS (RG)	Perception during chewing of fibrous particles of different shape and dimension
EGG (EG)	Aroma associated with egg (smell and flavour)
NUT (NU)	Aroma associated with baked chestnut (smell and flavour)
COOKED ODOR (CKO)	Olfactive impression associated with temperature treatment perceived via orthonasal.
BREAD CRUST (BC)	Aroma associated with bread crust (smell and flavour)
VEGETAL DRIED (VD)	Aroma associated with straw or dry leaves
SALTY (SA)	Taste associated with NaCl
BITTER (BI)	Basic taste produced by dilute aqueous solutions of various substances such as quinine or caffeine
AFTERTASTE (AT)	Taste and smell sensations that are perceived after swallowing
TASTE INTENSITY (TI)	Intensity of sensations perceived on the tongue
ASTRINGENT (AS)	Feeling factor associated with dryness and puckering of the mouth
HOMOGENEOUS TEXTURE (HT)	No residue, easy to chew
SHRIMP FLAVOUR (SF)	Aroma associated with shrimp cooked

Table S5. Sensory descriptors ranked by their level of discrimination on products, along with their corresponding *p*-values.

Descriptors	Test values	*p*-values
COLOR (CO)	5.813	0.000
SHRIMP FLAVOUR (SF)	5.447	0.000
BREAD CRUST (BC)	4.983	0.000
NUTS (NU)	4.612	0.000
PITTING (PI)	4.527	0.000
HOMOGENEOUS TEXTURE (HT)	3.799	0.000
ASTRINGENT (AS)	3.775	0.000
BITTER (BI)	3.604	0.000
VEGETAL DRIED (VD)	3.562	0.000
SURFACE ROUGHNESS (RG)	3.379	0.000
AFTERTASTE (AT)	2.986	0.001
EGG (EG)	2.685	0.004
SALTY (SA)	2.492	0.006
COOKED ODOR (CKO)	2.240	0.013
TASTE INTENSITY (TI)	1.402	0.080
